# Dimeric peroxiredoxins are druggable targets in human Burkitt lymphoma

**DOI:** 10.18632/oncotarget.6435

**Published:** 2015-11-30

**Authors:** Anna Trzeciecka, Szymon Klossowski, Malgorzata Bajor, Radoslaw Zagozdzon, Pawel Gaj, Angelika Muchowicz, Agata Malinowska, Anna Czerwoniec, Joanna Barankiewicz, Antoni Domagala, Justyna Chlebowska, Monika Prochorec-Sobieszek, Magdalena Winiarska, Ryszard Ostaszewski, Iwonna Gwizdalska, Jakub Golab, Dominika Nowis, Malgorzata Firczuk

**Affiliations:** ^1^ Department of Immunology, Medical University of Warsaw, Warsaw, Poland; ^2^ Institute of Organic Chemistry, Polish Academy of Sciences, Warsaw, Poland; ^3^ Department of Bioinformatics, Institute of Biochemistry and Biophysics, Polish Academy of Sciences, Warsaw, Poland; ^4^ Laboratory of Mass Spectrometry, Institute of Biochemistry and Biophysics, Polish Academy of Sciences, Warsaw, Poland; ^5^ Bioinformatics Laboratory, Institute of Molecular Biology and Biotechnology, Adam Mickiewicz University, Poznan, Poland; ^6^ Department of Hematology and Transfusion Medicine, Centre of Postgraduate Medical Education, Warsaw, Poland; ^7^ Laboratory of Experimental Medicine, Center of New Technologies, University of Warsaw, Warsaw, Poland; ^8^ Department of Diagnostic Hematology, Institute of Hematology and Transfusion Medicine, Warsaw, Poland; ^9^ Department of Pathology, Maria Sklodowska-Curie Memorial Cancer Center and Institute of Oncology, Warsaw, Poland; ^10^ Institute of Physiology and Pathology of Hearing, Warsaw, Poland; ^11^ Genomic Medicine, Medical University of Warsaw, Warsaw, Poland

**Keywords:** antioxidant enzymes, peroxiredoxin, therapeutic target, Burkitt lymphoma, thioredoxin

## Abstract

Burkitt lymphoma is a fast-growing tumor derived from germinal center B cells. It is mainly treated with aggressive chemotherapy, therefore novel therapeutic approaches are needed due to treatment toxicity and developing resistance. Disturbance of red-ox homeostasis has recently emerged as an efficient antitumor strategy. Peroxiredoxins (PRDXs) are thioredoxin-family antioxidant enzymes that scavenge cellular peroxides and contribute to red-ox homeostasis. PRDXs are robustly expressed in various malignancies and critically involved in cell proliferation, differentiation and apoptosis. To elucidate potential role of PRDXs in lymphoma, we studied their expression level in B cell-derived primary lymphoma cells as well as in cell lines. We found that PRDX1 and PRDX2 are upregulated in tumor B cells as compared with normal counterparts. Concomitant knockdown of PRDX1 and PRDX2 significantly attenuated the growth rate of lymphoma cells. Furthermore, in human Burkitt lymphoma cell lines, we isolated dimeric 2-cysteine peroxiredoxins as targets for SK053, a novel thiol-specific small-molecule peptidomimetic with antitumor activity. We observed that treatment of lymphoma cells with SK053 triggers formation of covalent PRDX dimers, accumulation of intracellular reactive oxygen species, phosphorylation of ERK1/2 and AKT and leads to cell cycle arrest and apoptosis. Based on site-directed mutagenesis and modeling studies, we propose a mechanism of SK053-mediated PRDX crosslinking, involving double thioalkylation of active site cysteine residues. Altogether, our results suggest that peroxiredoxins are novel therapeutic targets in Burkitt lymphoma and provide the basis for new approaches to the treatment of this disease.

## INTRODUCTION

Burkitt lymphoma (BL) is a relatively rare in Western countries, yet highly aggressive germinal B cell-derived malignancy. Intensive chemotherapy is the mainstay of treatment, however considerable drug toxicity prompts the need to develop novel less toxic and efficient approaches. One of the therapeutic challenges is extremely fast growth of BL – short doubling time and proliferation fraction of nearly 100%. Rapidly proliferating cells have been reported to have increased levels of reactive oxygen species (ROS) [1, 2], rendering these cells reliant on specific antioxidant enzymes [3, 4]. Several groups of enzymes account for red-ox balance, including peroxiredoxins (PRDXs) belonging to thioredoxin (TRX) family. Currently, little is known about the role of PRDXs in B cell-derived malignancies. Notably, PRDX1 expression was seen increased upon B cell activation and was high in lymphomas [5].

PRDXs are enzymes that utilize conserved Cys residues to catalyze peroxides reduction. In mammals, there are six PRDXs, classified depending on the number and localization of the catalytic Cys residues into 3 subtypes: typical 2-Cys (PRDX1–4), atypical 2-Cys (PRDX5), and 1-Cys (PRDX6) peroxiredoxins. For all subtypes, there are two steps in peroxide reduction. Firstly, the thiol of peroxidatic Cys attacks the peroxide oxygen and becomes oxidized to sulfenic acid. Secondly, the sulfenic acid is attacked by a resolving Cys thiol, and a disulfide bond is formed. In typical 2-Cys PRDXs, which are head-to-tail functional homodimers, the resolving Cys comes from the C-terminus of the other monomer. Atypical 2-Cys and 1-Cys PRDXs, with resolving Cys in the same subunit or coming from other proteins or peptides, respectively, are functional monomers. Finally, to complete the catalytic cycle, the disulfide bond formed in the PRDX active site is reduced by the TRX-TRXR-NADPH system [6, 7].

Hydrogen peroxide works as a second messenger; therefore, its generation and removal are strictly regulated. Although other groups of enzymes such as catalases and glutathione peroxidases also scavenge hydrogen peroxide, PRDXs are crucial antioxidants due to their abundance and prevalence in various cellular compartments. PRDXs also directly interact with a variety of key signaling proteins, such as MAPKs [8], c-ABL [9], c-MYC [10], or tyrosine phosphatase PTEN [11]. They protect redox-sensitive catalytic cysteine residues of phosphatases and suppress MAPK signaling and senescence [12].

The expression levels of PRDXs have been studied in a number of malignancies. For example, in lung [13], ovarian [14], and colorectal [15] cancers, high PRDX1 levels predict worse response to therapy. Yet, in a subtype of breast cancer PRDX1 is a biomarker of favorable prognosis [16]. Correspondingly, at the early stages of leukemogenesis, PRDX2 inhibits the proliferation of hematopoietic precursor cells [17]. On the other hand, PRDXs are highly expressed in promyelocytic leukemia cells and have cytoprotective, growth-promoting functions [18]. These findings highlight growing demand for small molecules targeting TRX-like enzymes as putative anti-tumor therapeutics. Such an example is adenanthin, a diterpenoid plant metabolite, which inhibits TRX-like enzymes and exerts anti-leukemic activity [18, 19].

We have recently designed a peptidomimetic inhibitor, SK053, aimed to bind TRX (compound 19a in [20]). This compound inhibits the activity of TRX and exerts cytostatic/cytotoxic effect with a clear preference towards rapidly proliferating cells, including murine (EMT6, Panc02, CT26) and human (Raji, Ramos, K562) tumor cell lines. Importantly, SK053 demonstrates antitumor activity in mice with no evidence of systemic toxicity [20]. In BL cell line Raji, SK053 induces endoplasmic reticulum stress-mediated apoptosis [21]. The inhibitor was designed to carry two electrophilic centers. The putative mechanism of the inhibitor binding to TRX involves a double thioalkylation of the two catalytic cysteine residues. In this study, using a biotin affinity probe-labeling approach, we identified typical 2-Cys PRDXs as targets for SK053 in human BL cell lines.

## RESULTS

### TRX-like antioxidant enzymes are upregulated in B cell-derived lymphoma primary cells and cell lines

**Figure 1:**
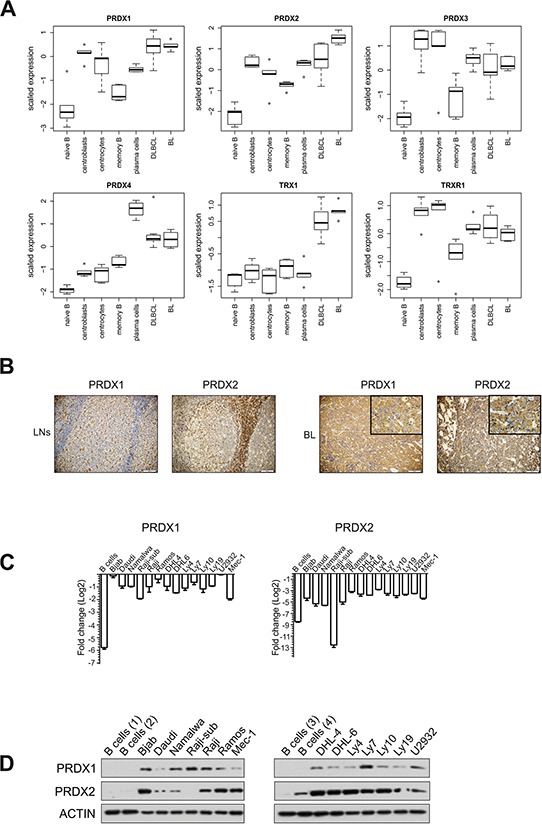
PRDX1 and PRDX2 are upregulated in lymphoma cells **A.** Analysis of the expression of PRDX1-4, TRX1, TRXR1, in the data set E-GEOD-12453 deposited in the ArrayExpress database. The expression is analyzed in 5 sub-populations of normal B cells (left-hand) and in malignant B-cell derived DLBCL and BL cells (right-hand). **B.** IHC staining of lymph nodes (LNs) with reactive follicular hyperplasia (left panel) and representative BL cells for PRDX1 and PRDX2 (EnVision stain, 20x). In the right panel, insertions in the upper right corners have higher magnification (60x) of BL tissue stained with anti-PRDX1 and PRDX2, respectively. **C.** qRT-PCR analysis of the PRDX1 and PRDX2 gene expression in B cell-derived tumor cell lines and four CD19+ control B cells. qPCR data were normalized to housekeeping genes, RPL29 and β-2-microglobulin. The fold change is displayed on a log2 scale. Mean values from two independent experiments are shown, the bars indicate the standard error of the mean (SEM). **D.** Representative Western blot for the PRDX1 and PRDX2 protein levels in B cell-derived tumor cell lines and CD19+ control B cells obtained from 4 healthy donors.

To gain deeper insight into the PRDX-TRX-TRXR antioxidant system in B cell malignancies, we analyzed the deposited microarray data [22] on the expression of PRDX1–4, TRX1, and TRXR1. The data contained malignant B cells derived from BL and diffuse large B-cell lymphoma (DLBCL) patients and normal B cell subsets. We observed upregulated expression of PRDX1, PRDX2, and TRX1 in malignant cells. PRDX2 expression was extremely high in BL (Figure 1A, Supplementary Tables S1 and S2). Considering that TRX1 is already a known therapeutic target in lymphoma [23], we focused on two PRDX cytoplasmic isoforms, PRDX1 and PRDX2. Immunohistochemistry of primary BL revealed robust membrane and cytoplasmic staining for both PRDX1 and PRDX2 (Figure 1B). To extend these observations, we analyzed the expression of PRDX1 and PRDX2 in a panel of B cell-derived tumor cell lines. In the majority of cell lines, both PRDX1 and PRDX2 were highly expressed, as compared with normal B cells (Figure 1C, 1D Supplementary Figure S1). Altogether, the above results demonstrate increased expression of TRX-like antioxidant enzymes in lymphoma cell lines as well as in primary cells from lymphoma patients.

### Downregulation of PRDX1 and 2 decreases the proliferation and survival of lymphoma cells

**Figure 2:**
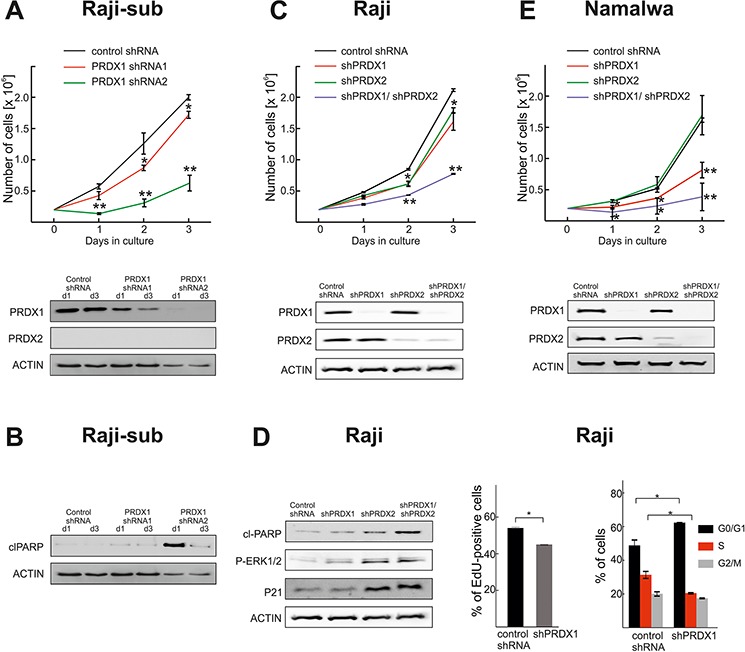
Both PRDX1 and PRDX2 control the proliferation and survival of lymphoma cells **A.** Raji-sub cells were treated with lentiviruses encoding two different PRDX1-specific shRNAs or control, non-targeting shRNA. After puromycin selection, the number of viable cells was evaluated in a hemocytometer for three consecutive days. At days 1 (d1) and 3 (d3) cells were collected, and the levels of PRDX1, PRDX2 were evaluated by immunoblotting. **B.** The level of cleaved PARP (clPARP) was assessed by immunoblotting. **C.** Raji cells were subjected to transduction with lentiviruses carrying PRDX2-specific shRNA and hygromycin resistance gene. After 6 days of antibiotic selection, the cells were infected with lentiviruses encoding PRDX1-targeting shRNA2 and puromycin resistance genes. Following 3 days of puromycin selection, the number of viable cells was assessed with flow cytometry, by counting the PI-negative cells. Three days after puromycin selection, cells were collected and the levels of PRDX1 and PRDX2 were analyzed by immunoblotting. **D.** Levels of clPARP, P-ERK1/2, p21 were assessed at day 3 post selection by immunoblotting. DNA synthesis was assessed in Raji cells expressing PRDX1-specific shRNA2 (shPRDX1) and control cells expressing non-targeting shRNA (control shRNA) with the Click-iT EdU (5-ethynyl-2′-deoxyuridine) incorporation assay. Data show the % of EdU-positive cells, mean values from two independent repeats ± SD, *P < 0.05. The cell cycle distributions in Raji cells expressing PRDX1-specific shRNA2 (shPRDX1) and control cells expressing non-targeting shRNA (control shRNA) were evaluated with a propidium iodide flow cytometry-based assay. The error bars indicate the SD (*n* = 2), *P < 0.05. **E.** Namalwa cells were subjected to sequential lentiviral transductions to downregulate PRDX1 and PRDX2, as described in **C.** and the number of viable cells was assessed in a hemocytometer for three consecutive days. The degree of PRDX1 and PRDX2 knockdown was assessed by immunoblotting in cells collected 3 days after puromycin selection.

Next, we studied the effects of PRDX1 downregulation on the growth rate and survival of BL Raji cells which express PRDX2, and of a Raji subline, (Raji-sub) [24], which we identified not to express PRDX2. In Raji-sub cells, partial reduction of the PRDX1 level (shRNA1) slightly reduced the growth rate, while more pronounced inhibition of PRDX1 expression (shRNA2) resulted in a significant inhibition of cell growth (Figure 2A) and the induction of apoptosis, as measured by PARP cleavage (Figure 2B). In Raji cells the silencing of a single PRDX1 or PRDX2 gene slightly diminished cell proliferation. However, the effects of concomitant PRDX1 and PRDX2 knockdown resulted in ERK1/2 phosphorylation, an increase in p21, and the induction of apoptosis (Figure 2C, 2D). Moreover, in Raji cells with a reduced PRDX1 level, we observed decreased DNA synthesis and G0/G1 cell cycle arrest (Figure 2D). In Namalwa, another BL cell line, PRDX1, but not PRDX2, knockdown alone already significantly reduced cell growth rate. Importantly, the growth was further blunted in cells with simultaneous PRDX1 and PRDX2 knockdown. As in Raji cells, PRDX1 downregulation reduced the rate of DNA synthesis (Supplementary Figure S2). Altogether, these results demonstrate that concomitant PRDX1 and PRDX2 knockdown significantly attenuates growth rate of BL cells.

### PRDX1 is a target for SK053

**Figure 3:**
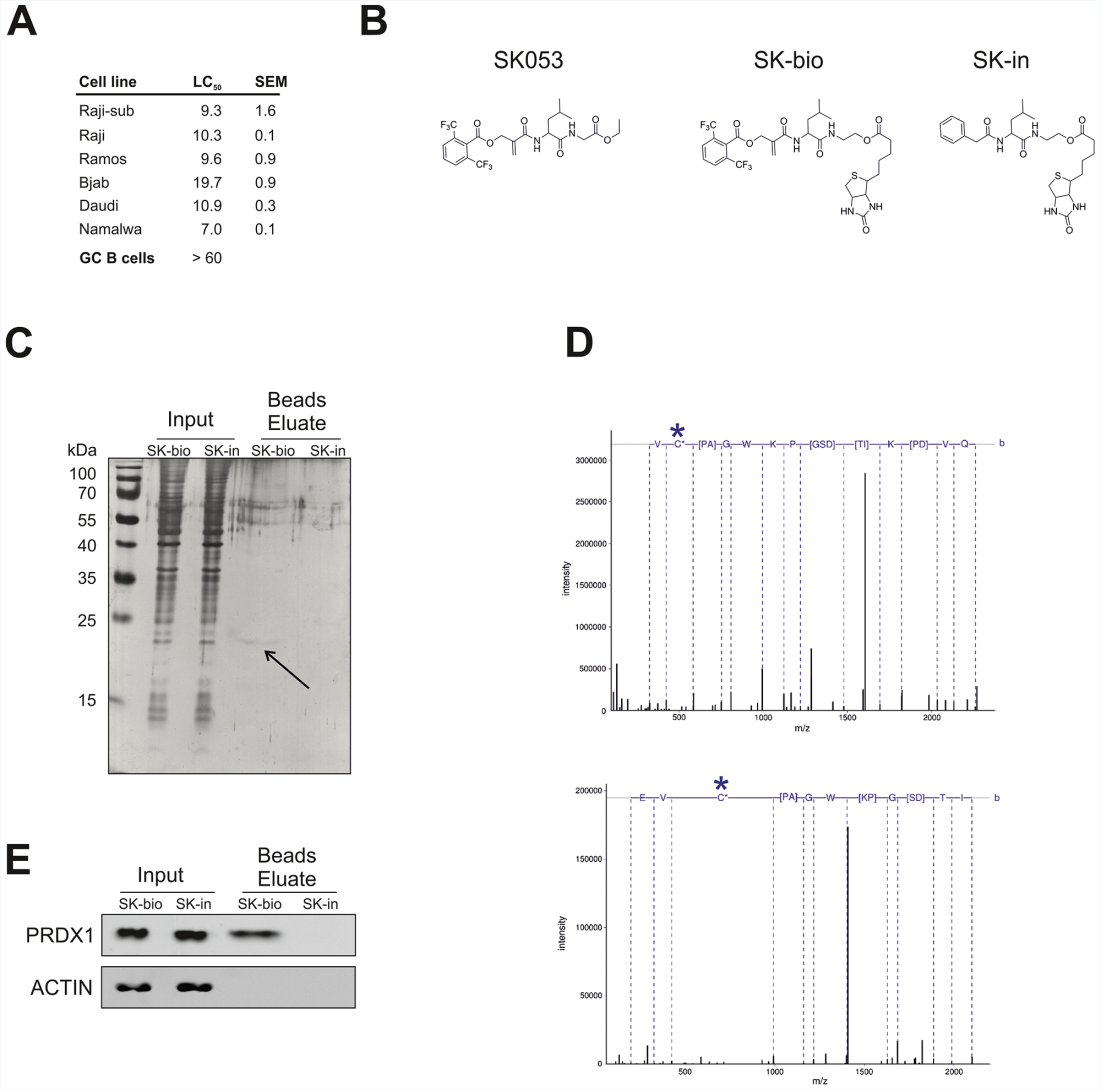
SK053 covalently binds to PRDX1 in Raji cells **A.** Cytostatic/cytotoxic effects of SK053 on human BL cell lines and normal germinal center B cells (GC B cells). BL cell lines were incubated with SK053 for 48 h and subjected to a MTT viability assay. The LC_50_ was calculated in Graphpad Prism 5 by nonlinear regression dose-response analysis with variable slopes. The SEM was calculated based on two independent experiments. GC B cells isolated from human tonsils (*n* = 3) were isolated and cultured as described in Methods. Number of viable cells after 48 h treatment with SK053 was assessed using Muse™ Cell Analyzer (Merck Millipore). LC_50_ was calculated in Graphpad Prism 5, as described above for BL cell lines. **B.** Chemical structure of SK053, its biotinylated derivative SK-bio, and the inactive biotinylated analog devoid of the electrophilic center, SK-in. **C.** Raji-sub cells were incubated with SK-bio or SK-in for 2 h, lysed, and biotin-labeled proteins were affinity-purified on avidin-coated beads. Total protein was resolved by SDS-PAGE and visualized by silver staining. The arrow indicates the band that was excised and identified by mass spectrometry. **D.** Tandem mass spectra of the Cys-173-containing peptide, HGEVCPAGWKPDGSDTIKPDVQK. The site of cysteine modification is marked with a star. The upper panel spectrum corresponds to a peptide modified with iodoacetamide (+57.021), with the parent ion m/z 802.731 and a charge 3+. The bottom panel presents the spectrum of a peptide in which cysteine bears an inhibitor (+466.225), with parent ion m/z 704.600 and a charge 4+. **E.** The same samples as in **C.** were subjected to immunobloting using antibodies specific to PRDX1 and β-actin (ACTIN).

Considering the elevated levels of TRX-like enzymes as well as their pro-survival role in lymphoma cells, we searched for candidate compounds for their pharmacologic inhibition. We have previously reported on the synthesis of the thiol-specific small molecule peptidomimetic with antitumor activity, SK053. Here, we have found that BL cell lines are sensitive to SK053, with an LC_50_ ranging from 7 μM for the Namalwa up to almost 20 μM for Bjab cells. Importantly, normal germinal center B cells (GC B cells) isolated from human tonsils were more resistant to SK053 (LC_50_ > 60 μM), indicating selectivity towards malignant B cells (Figure 3A).

To identify targets for SK053 in BL cells, we synthesized biotin-tagged derivative of SK053 (SK-bio) and an inactive, biotinylated analogue that lacks the electrophilic double bond (SK-in), which was used as a negative control (Figure 3B, Supplementary Figure S3). Only the active, SK-bio preserved cytostatic/cytotoxic activity (Supplementary Figure S4). A band of approximately 20 kDa was detected in a silver-stained gel only for cells incubated with active SK-bio (Figure 3C). The protein was identified by MS as PRDX1, with > 90% of sequence coverage. Furthermore, in a collection of tryptic peptides, we searched for a modification of 540 Da, corresponding to the mass of SK053, after the first addition reaction, and the modification of 466 Da, which corresponds to the part of SK053 after the addition and elimination of the leaving group, according to the previously described mechanism (Scheme 3 in [20]). We found the tryptic peptide, containing Cys173, with a mass modification of 466 Da. The fragmentation of the peptide confirmed that the modification is on Cys173 (Figure 3D). Immunoblotting revealed that PRDX1, but neither TRX1 nor TRXR1, is present in the beads eluates from cells treated with SK-bio (Figure 3E, Supplementary Figure S5). All of the above results demonstrate that SK053 covalently binds to PRDX1 in Raji cells.

### SK053 crosslinks typical 2-Cys PRDXs dimers

**Figure 4:**
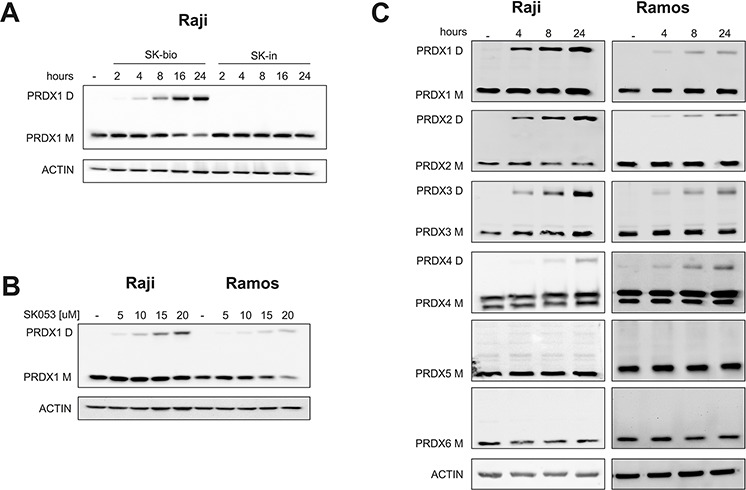
SK053 triggers the formation of covalent typical 2-Cys PRDXs dimers Cells were cultured with SK053, biotinylated SK053 (SK-bio) or its inactive counterpart (SK-in), harvested and analyzed by immunoblotting using specific antibodies, as indicated on the left side of each blot. Bands labeled M migrated at ∼ 22 kDa, a mass corresponding to the molecular weight of the peroxiredoxin monomer, and bands labeled D migrated at ∼ 44 kDa, a mass corresponding to the molecular weight of the peroxiredoxin dimer. **A.** The concentration of SK-bio and SK-in was 55 μM (=LC_80_). **B.** Cells were cultured with the indicated concentrations of SK053 for 24 h. **C.** 10 μM of SK053 was used, which is close to the LC_50_.

Next, we monitored the levels of PRDX1 in Raji cells after SK-bio treatment. As shown in Figure 4A, we observed a time-dependent accumulation of the additional protein band recognized with anti-PRDX1 antibody, with a mass corresponding to PRDX1 dimer. The band was also observed in cells treated with unlabeled SK053 (Figure 4B). As the protein lysates were resolved under denaturing and reducing conditions, the above results suggest that the higher-molecular weight band is covalent and not disulfide-linked. To further expand on this observation, we tested the levels of monomers and dimers of all six PRDXs in Raji and Ramos cells that were treated with SK053. As presented in Figure 4C, the dimers could only be detected for PRDX1–4, which are functional dimers. Treatment with other thiol-reactive compounds, adenanthin and AW464, did not trigger dimer formation, suggesting that it is a unique feature of SK053 (Supplementary Figure S6).

### SK053 crosslinks PRDX1 dimers via active site cysteine residues

**Figure 5:**
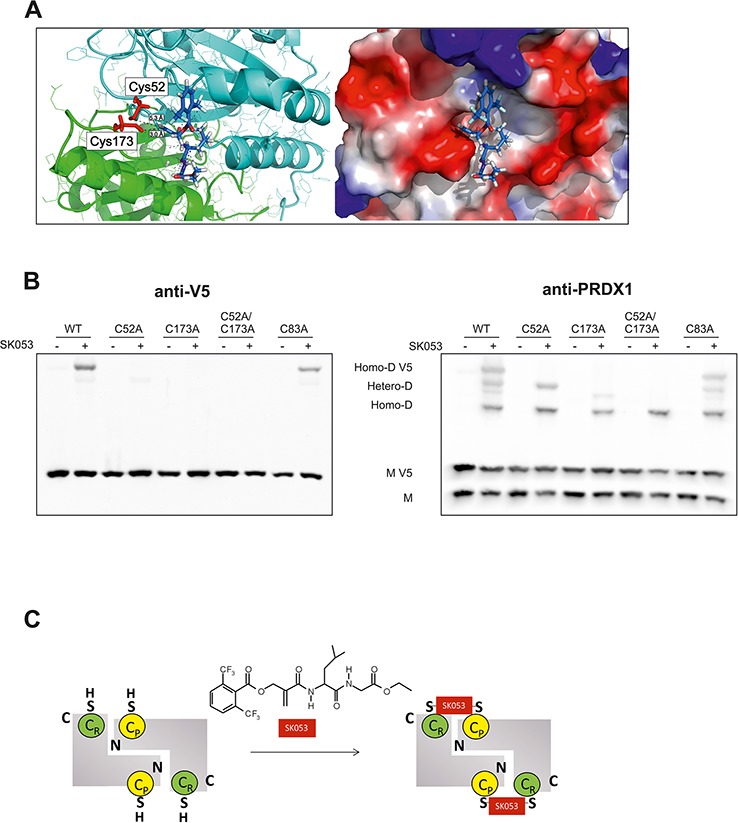
SK053 cross-links PRDX1 monomers via its active site cysteine residues, Cys52 and Cys173 **A.** Docking of the SK053 molecule to the binding pocket around Cys173 of the rat PRDX1 model (PDB code 1QQ2), in which all rat-specific amino acids were substituted to human-specific counterparts. Active site Cys residues, Cys52 and Cys173, are indicated in red. Distances between the sp2 carbon and Cys thiols are indicated with dashed black lines. Favorable hydrogen bonds are shown as blue dashed lines. **B.** HEK293T cells were transfected with a pLenti6-PRDX1-V5 plasmid encoding a V5-tagged, wild-type protein (WT), or protein variant with selected cysteines mutated to alanines. Twenty-four hours after transfection, 41 μM (∼ LC_50_) SK053 (+) or DMSO (−) was added, and after an additional 24 h, the cells were harvested and analyzed by immunoblotting using anti-V5 and anti-PRDX1 antibodies. Note that on the PRDX1 immunoblot (right panel), the highest molecular weight band corresponds to the V5-tagged PRDX1 homodimer (Homo-D V5), the lowest to the untagged homodimer (Homo-D) and the two middle bands to the heterodimers (Hetero-D). **C.** Scheme presenting double thioalkylation of the active site Cys residues of dimeric 2-Cys PRDXs, resolving Cys (C_R_) and peroxidatic Cys (C_P_) with SK053.

The above results imply that SK053 binds to PRDX1–4 in lymphoma cells. In agreement, SK053 inhibited PRDX1 activity and SK-bio bound recombinant human PRDX1 monomer and triggered dimer formation, however less efficiently than in cells (Supplementary Figure S7). To gain more insight into the binding of SK053 to PRDX1, we docked SK053 to the PRDX1 model. During the catalytic cycle, the regions bearing active site Cys residues undergo substantial conformational rearrangements ([25, 26], Supplementary Figure S8A). Assuming that SK053 covalently binds to PRDX1 thiols, we first docked the inhibitor to the reduced PRDX1 variant [25]. However, in this conformation, the active site Cys residues are not easily accessible to the bulky inhibitor (data not shown). In contrast, the inhibitor fits well into the oxidized form [27]. The inhibitor docked with the best scores in the shallower pocket in the region around the resolving Cys173 (Figure 5A, Supplementary Figure S8B).

Considering the accumulation of covalent PRDX1 dimers upon SK053 in lymphoma cells, we speculated that the two catalytic Cys residues, Cys52 and Cys173, are both binding sites of SK053. To test this hypothesis, we generated constructs for the expression of V5-tagged human wild-type PRDX1 and a series of point mutated variants, in which Cys residues were substituted with Ala, including single catalytic Cys residues (C52A, C173A), double catalytic Cys (C52A/C173A), and the variant C83A, which has a mutation of the Cys residue outside the active site. The mutation of each of the catalytic Cys, but not the Cys83, abolished the formation of the V5-tagged homodimer, as detected with anti-V5 antibody (Figure 5B, left panel). Although heterodimers of V5-tagged PRDX1, and its native untagged form, could be detected for single active site Cys mutants, they were absent in the case of the double C52A/C173A mutant. C83A variant behave like wild-type (Figure 5B, right panel). These results confirmed the essential role of the two catalytic Cys residues in SK053-triggered crosslinking of PRDX1 dimers and allowed us to propose the mechanism of double thioalkylation of peroxidatic and resolving cysteines, presented in Figure 5C. We presume that similar mechanism occurs for all typical 2-Cys PRDXs.

### SK053 triggers ROS accumulation, growth arrest and apoptosis

**Figure 6:**
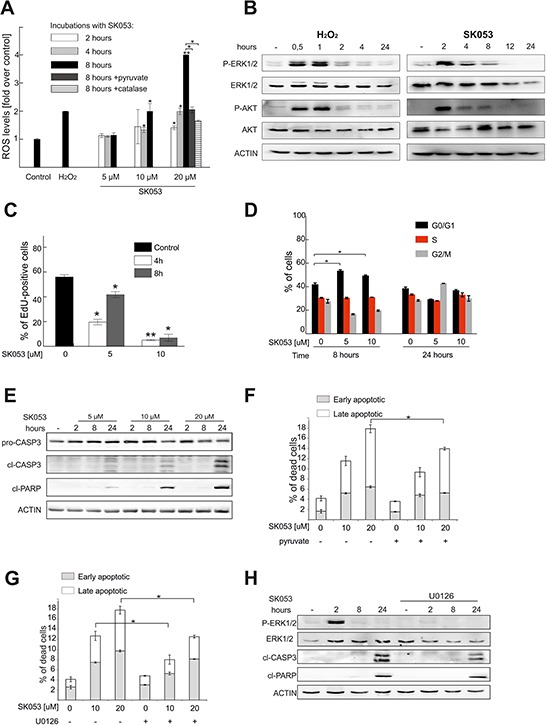
SK053 triggers ROS accumulation, cell cycle arrest and apoptosis **A.** Raji cells were loaded with CM-H2-DCFDA dye and treated with SK053 for the indicated times or for 2 h with 100 μM H_2_O_2_, as a positive control. Two additional groups were included, in which Raji cells were pre-treated with 2 mM pyruvate (dark grey bar) or 100 μg/ml catalase (bar filled with horizontal lines) for 30 min and then exposed to 20 μM SK053 for 8 h. The green fluorescence intensity was evaluated by flow cytometry, and the ROS levels are presented as the fold change over untreated control. The mean values from two independent repeats ± SD are shown, *P < 0.05, **P < 0.001. **B.** Immunoblotting of Raji cells treated with 10 μM SK053 or 50 μM hydrogen peroxide for the indicated time. **C.** The rate of DNA synthesis was assessed with the Click-iT EdU incorporation assay. Data show the % of EdU-positive cells, mean values from two independent repeats ± SD, *P < 0.05, and **P < 0.001 versus untreated control cells. **D.** The cell cycle distribution after treatment of Raji cells with SK053 was analyzed using a propidium iodide flow cytometry-based assay. The error bars indicate the SD (*n* = 2), *P < 0.05 versus untreated control cells. **E.** Raji cells were treated with SK053 for the indicated time, lysed and analyzed by immunoblotting. **F.** Apoptosis was evaluated in Raji cells treated for 24 h with SK053, or pre-treated with 2 mM pyruvate for 30 min and then incubated with SK053 for 24 h. The numbers of early and late apoptotic cells were assessed using annexin V and propidium iodide staining, followed by flow cytometry analysis. The mean values are presented ± SD (*n* = 2), *P < 0.05. **G.** Raji cells were treated and analyzed as described in panel **F.** but 30 μM U0126 was used for pre-treatment, instead of pyruvate. **H.** Immunoblotting of Raji cells treated with SK053 +/− U0126.

As SK053 inhibits PRDX1 catalytic activity, we next investigated the effects of SK053 on ROS homeostasis. As shown in Figure 6A and Supplementary Figure S9, SK053 increased the levels of ROS in Raji cells in a time- and dose- dependent manner. Importantly, the accumulation of ROS was attenuated in cells that were pre-treated with pyruvate, a non-enzymatic hydrogen peroxide scavenger [28], as well as with catalase, an enzyme specifically removing hydrogen peroxide (Figure 6A). These data imply that hydrogen peroxide is a dominant ROS accumulated in cells incubated with SK053.

It is established that hydrogen peroxide induces phosphorylation of kinases, such as ERK1/2 and AKT [11, 12]. Indeed, in our study, treatment of Raji cells with SK053 triggered prolonged ERK1/2 and AKT phosphorylation (Figure 6B). It was previously reported that increased ROS due to PRDXs downregulation induced cell cycle arrest [29, 30]. Accordingly, SK053 significantly reduced DNA synthesis (Figure 6C) and triggered cell cycle arrest (Figure 6D). In addition, SK053 induced apoptosis, as evidenced by the occurrence of cleaved caspase-3 and PARP (Figure 6E) and the increase in the numbers of early and late apoptotic cells (Figure 6F). Pretreatment with pyruvate or ERK1/2 phosphorylation inhibitor, U0126, attenuated SK053-induced apoptosis (Figure 6F–6H). Taken together, these results demonstrate that SK053 inhibits cell proliferation, and, at higher doses, triggers apoptosis. Moreover, SK053-induced cell death is, at least to some extent, triggered by ERK1/2 signaling.

## DISCUSSION

In this work, we identify PRDXs as a new, attractive therapeutic targets in BL. Particularly, we show that PRDX1 and PRDX2 are abundantly expressed in B cell-derived primary lymphoma cells and cell lines as well as that they promote lymphoma cell proliferation and survival. Moreover, we identify 2-Cys PRDXs as molecular targets for a new thiol-targeting compound, SK053, which confirms druggability of PRDXs in lymphoma cells. We would like to emphasize that SK053, as a peptidomimetic, is not an optimal small molecule for further pursuing towards clinical trials. However, our mechanistic investigations provide evidence that double thioalkylation of active site cysteines is a novel, effective strategy to inhibit dimeric PRDXs and may guide the design of a new class of anti-lymphoma therapeutics.

Peroxiredoxins have been studied in different tumors; however, their roles vary [8, 17]. Interestingly, downregulation of TRX inhibitory protein VDUP1, and upregulation of PRDX3 and PRDX4, correlated with poor prognosis of DLBCL patients, indicating tumor-promoting role of TRX-like enzymes in lymphomas [31]. Our results support this finding. Specifically, we demonstrate that PRDXs are highly expressed in human tumor B cell-derived primary lymphoma cells and cell lines (Figure 1, Supplementary Figure S1, Supplementary Tables S1 and S2). Furthermore, in a model of BL cell lines, we show that concomitant downregulation of PRDX1 and PRDX2 diminishes the growth rate of lymphoma cells (Figure 2, Supplementary Figure S2). These observations are in agreement with previously published data that PRDX knockdown decreases cell proliferation and triggers cell cycle arrest and senescence in murine embryonic cells as well as in human glioma cells [29, 30, 32]. All of these findings provide a new information that, in BL cells, both PRDX1 and PRDX2 promote cell growth and proliferation, with a functional overlap between these two enzymes.

Lymphoma cells are characterized by a rapid proliferation rate and increased levels of ROS, therefore they require strict control of red-ox homeostasis [1]. Dozens of enzymes account for red-ox homeostasis, and some of them are redundant. Recent studies revealed that deletion of one antioxidant enzyme triggers compensatory upregulation of the other and that inhibition of both glutathione and TRX-based antioxidant systems synergistically kills cancer cells [4]. Here we present that the small-molecule inhibitor, SK053, which was previously shown to inhibit TRX-TRXR system, has broader specificity towards other TRX-like proteins. Using the biotin affinity labeling approach, we identified dimeric 2-Cys PRDXs as targets for SK053 (Figure 3). We did not succeed to isolate TRX1 and TRXR1 by biotin affinity labeling as covalent SK053 targets (Supplementary Figure S5). Possibly, their binding may not be preserved during the isolation and separation of biotinylated proteins. Indeed, using a different procedure, involving the incubation of SK-bio with Raji cell lysates, we could isolate both TRX1 and TRXR1 as SK-bio-binding proteins (Supplementary Figure S10); however, we consider the latter approach less physiologically relevant. The affinity labeling has proven effective for a small-molecule target identification in multiple instances [18, 33]; nevertheless, it has some limitations. Therefore, we present evidence from additional experiments confirming that dimeric 2-Cys PRDXs are SK053 targets in lymphoma cells. Foremost, only PRDX1-4, which are functional dimers, undergo dimer crosslinking upon treatment with SK053. The dimer formation is dose-dependent and proceeds in the time during incubation with SK053, suggesting that the inhibitor is preserved for up to 24 h in cells (Figure 4A). Moreover, we show that double thioalkylation occurs at the active site Cys residues (Figure 5), implying that SK053 blocks PRDX enzymatic activity. Indeed, SK053 binds to recombinant human PRDX1 and inhibits its activity in an *in vitro* assay (Supplementary Figure S7). Consistently, in Raji cells SK053 triggers ROS accumulation, ERK1/2 and AKT phosphorylation, kinase signaling that is typically induced in response to hydrogen peroxide (Figure 6A, 6B Supplementary Figure S9). At a lower concentration (5 μM), SK053 causes reversible inhibition of DNA synthesis and cell cycle arrest (Figure 6C, 6D). At concentrations above 10 μM, SK053 abrogates DNA synthesis and triggers apoptosis (Figure 6E, 6F). Interestingly, pretreatment with the inhibitor of ERK1/2 activator, U0126, blocks the phosphorylation of ERK1/2 and attenuates SK053-induced apoptosis, implicating a role for ERK1/2 signaling in SK053-induced lymphoma cell death (Figure 6G, 6H). Canonically, Ras-Raf-MEK1-ERK1/2 signaling stimulates tumor cell proliferation; however, it may also mediate cell death, including oxidative stress-induced apoptosis [34].

SK053, unlike other thiol-reactive inhibitors adenanthin and AW464, triggers crosslinking of PRDX1–4 dimers (Supplementary Figure S6). Our studies with V5-tagged PRDX1 revealed that the thioalkylation reaction involves catalytic cysteines (Figure 5). Indeed, these residues approach one another to form a disulfide bond in the course of catalysis. During the catalytic cycle, peroxiredoxins undergo substantial conformational rearrangements, particularly within the loop bearing resolving Cys (Supplementary Figure S8A). Our attempts to dock SK053 to the rat PRDX1 imply that the first thioalkylation occurs at the resolving Cys173. Intact SK053 fits better in the shallower pocket around Cys173. In the model with SK053 docking to the PRDX1 dimer, the unsaturated carbon localizes at a distance of 3 Å from the Cys173 sulfur. In addition, the aromatic acyloxy group is placed in a positively charged environment, which can support its dissociation and formation of the second electrophilic center (Figure 5A). Accordingly, MS analysis of tryptic peptides derived from the biotin affinity purified PRDX1 revealed the modification of the resolving Cys by the mass of 466 Da, which corresponds to the inhibitor part after the elimination of the acyloxy group (Figure 3C). It is not entirely clear how the second thioalkylation occurs. In lymphoma cells, the crosslinked dimers are barely detectable after 2 h of incubation with SK053 and they gradually accumulate over a period of at least 24 h, showing that the process is slow in cells (Figure 4) and even less efficient *in vitro* (Supplementary Figure S7B). This may indicate that structural rearrangements, which may also involve partner protein interactions, are necessary for the second thioalkylation step. This is plausible, especially considering increased oxidative stress in cells treated with SK053. Structural studies have revealed that oxidation of peroxidatic Cys triggers local unfolding and major structural rearrangements of the regions around catalytic cysteines [25]. All these results demonstrate that double thioalkylation of catalytic cysteines is a novel strategy to inhibit dimeric PRDXs and provide a mechanistic basis for the design of a new class of dimeric 2-Cys PRDXs inhibitors.

In summary, we present that lymphoma cells abundantly express TRX-like antioxidant proteins. Furthermore, we demonstrate that peptidomimetic small-molecule SK053 covalently binds dimeric 2-Cys PRDXs in human BL cell lines. Importantly, our findings indicate that PRDX1 and PRDX2 have complementary growth-supporting functions in a BL model *in vitro*. Based on these findings, we conclude that targeting TRX-like antioxidant proteins may be considered a promising approach to therapy of BL. At the same time, our results suggest that targeting a single PRDX may be insufficient in this disease and pinpoint the need for the development of pan-PRDX inhibitors with a prospective therapeutic use.

## MATERIALS AND METHODS

### Chemicals

SK053 was synthesized as described in [20], AW464 (7a) as described in [35]. The synthesis of biotinylated SK053 analogue (SK-bio) and its inactive counterpart (SK-in) is described in the Supplementary Methods. Adenanthin was purchased from Faces Biochemical Co., Wuhan, China and U0126 from Selleckchem, USA.

### Cell culture

Human BL cell lines (RAJI, Ramos, BJAB, Daudi, and Namalwa), B-cell chronic lymphocytic leukemia (Mec-1), DLBCL (DHL-4, DHL-6, Ly4, Ly7, Ly10, Ly1, and U2932) and human embryonic kidney (HEK293T) cells were purchased from ATCC. The Raji-sub cell line is a complement-sensitive sub-strain of Raji [24]. The authentication of the Raji-sub cell line was confirmed by genotyping (IdentiCell, Denmark). Cells were cultured in DMEM (HEK293T) or RPMI-1640 medium (all other cell lines) supplemented with 10% heat-inactivated fetal bovine serum and antibiotic at 37°C, 5% CO_2_, in a humidified atmosphere. In all experiments, control groups were treated with vehicle (DMSO).

### Isolation and culture of germinal center B cells

Human tonsils were derived from children undergoing tonsillectomy (*n* = 3). Their use was approved by the Ethics Committee at the Institute of Physiology and Pathology of Hearing, Warsaw, Poland. After resection, tonsils were cut into small pieces and incubated in the RPMI medium supplemented with collagenase and DNase for 30 min at 37°C with gentle shaking and filtered through 70 μm cell strainer. After washing in PBS, mononuclear cells were isolated with Histopaque 1077 density gradient and subjected to CD19 negative selection using EasySep™ Human B Cell Enrichment Kit (STEMCELL Technologies). The isolated B cells were stained with anti-CD20-PE and anti-CD38-FITC antibodies and CD20+/CD38+ germinal center B cells (GC B cells) were separated by fluorescence-activated cell sorting using FACSAria III.

For co-culture experiments, HT-1080 cells expressing human CD40L were incubated with mitomycin C (10 μg/ml, Sigma Aldrich) for 3 h, and seeded into 96-well plates, 1 × 10^4^ cells per well. Next day, when HT-1080 cells were attached, the medium was removed and GC B cells were added, 1 × 10^5^ cells per well, in RPMI full medium supplemented with 25 ng/ml IL-21 (Peprotech). To assess SK053 cytostatic/cytotoxic effects, cells in co-culture were incubated with SK053 in a concentration range 5–80 μM for 48 h. The numbers of viable cells were assessed with Muse™ Cell Analyzer, using Count &Viability Reagent (Merck Millipore). The LC_50_ was calculated in Graphpad Prism 5 by nonlinear regression dose-response analysis with variable slopes.

### Affinity isolation of biotinylated proteins

Raji-sub or Raji cells were cultured in a serum-free RPMI medium in the presence of 100 μM SK-bio or SK-in, with 2 × 10^7^ cells in each group. After two hours, the cells were harvested, lysed, and biotinylated proteins were isolated as described in [19]. The protein band that migrated around 20 kDa was excised from the gel and analyzed by MS. Alternatively, the eluates were subjected to immunoblotting.

### Mass spectrometry

LC-MS analysis of gel slices was conducted as described in [36]. The raw data were pre-processed with Mascot Distiller software (v. 2.3, Matrix Science) and obtained peak lists were searched against the database of human protein sequences from SwissProt combined with its randomized version (40464 sequences) using Mascot search engine (version 2.4, 8-processors onsite license) (Matrix Science) with the following search parameters: enzyme specificity – semi-trypsin, missed cleavages – 1, variable modifications – oxidation (M), carbamidomethylation (CK), SK053(C), peptide mass tolerance – ± 20 ppm, fragment mass tolerance – ± 0.6 Da. Decoy option in Mascot was activated in order to assure the false discovery rate below 1%, and the resulting Mascot score threshold of 63 was applied to results.

### MTT assay

Cells were seeded into 96-well plates at a density of 2 × 10^4^ per well and treated with investigated compounds in a final volume of 200 μl. After 48 hours, MTT assay was performed as described in [21].

### ROS assessment

Cells were loaded with CM-H2-DCFDA (Molecular Probes, USA) according to manufacturer's protocol, using 1 μM dye concentration for 30 min at 37°C. Next, labeled cells were incubated with SK053 or H_2_O_2_ in a culture medium at 37°C, washed in PBS and the intensity of green fluorescence was analyzed in BD Accuri C6 Flow Cytometer (BD Biosciences, USA).

### Immunoblotting

Cells were subjected to drug treatment at a density of 0.1–0.2 mln/ml. Cell lysis, protein concentration measurement and immunoblotting was performed as described in [21]. Antibodies used: Cell Signalling: cleaved PARP - 5625, caspase 3 - 9662, p21 Waf1/Cip1 – 2947, biotin HRP-linked – 7075, Akt1 - 2967, phospho-Akt (Thr308) – 2965, phospho-p44/42 MAPK (P-ERK1/2) (Thr202/Tyr204) – 9101, p44/42 MAPK (ERK1/2) – 9107, TRX1 - 2429; Sigma-Aldrich: PRDX1 - HPA007730, PRDX6 - HPA006983, β-actin-HRP – A3854; Abcam: PRDX2 - EPR5155; AbFrontier: PRDX3 - LF-MA0044, PRDX4 - LF-MA0014, PRDX5 - LF-MA0002, Life Technologies: V5 - R96025; Santa Cruz Biotechnology Inc.: TRXR1 – sc-20147, Enzo Life Sciences: CRT - ADI-SPA-600.

### Site-directed mutagenesis

The cysteine-to-alanine mutations were introduced to pLenti6/V5-DEST-PRDX1 using QuikChange XL Site-Directed Mutagenesis Kit (Agilent Technologies, USA) according to manufacturer's instruction. The primers are provided in Supplementary Table S3. Mutations were confirmed by DNA sequencing.

### DNA synthesis

To evaluate the rate of DNA synthesis, for the last hour of incubation with SK053, 5-ethynyl-2′-deoxyuridine (EdU) was added to a final concentration of 50 μM. Next, cells were fixed, permeabilized and labeled according to Click-iT^®^ EdU Alexa Fluor^®^ 488 Imaging Kit protocol (Life Technologies, USA) and the number of EdU-positive cells was assessed by flow cytometry.

### Cell cycle

Cells treated with SK053 were washed with PBS and fixed in 70% ethanol at −20°C overnight. Next, cells were washed twice with PBS, and incubated in propidium iodide (50 μg/ml) containing 200 μg/ml RNase A at 37°C for 30 min. DNA content was analyzed by flow cytometry and FlowJo software. Cell cycle distribution was determined from 10^4^ cells using Watson mathematical model with constraints set on the vehicle control (DMSO). The obtained results were further normalized using below formula so percentages of cells in each phase add up to 100%: normalized proportion of a phase= (proportion of a phase/sum of all proportions) x 100%.

### Apoptosis

Apoptosis was evaluated using Annexin V Apoptosis detection kit (eBioscience, USA), according to manufacturer's protocol.

### Real-time PCR

The RNA extraction, cDNA isolation and RT-PCR assay was performed as previously described [16].

### Modeling and ligand docking

PRDX1 models with point mutations were prepared based on homology modeling procedures, using Modeller [37] and evaluated with MetaMQAP [38] and PROQ [39]. Protein structures superpositions were prepared with Swiss-PDBViewer [40]. Figures were prepared with PyMOL 1.5 (Schrödinger). For docking procedures we selected crystal structures-derived models (PDB codes 1QQ2 and 2Z9S) and corresponding models with point mutations, with all rat-specific residues substituted with human equivalents. Docking simulations were carried out with Surflex-Dock 2.6 software [41] based on the “anchor-and-grow” algorithm for optimized structures of small molecules. We used elNémo web server [42] to calculate proteins normal modes. Files with coordinates are available in Supplementary Data.

### Immunohistochemistry (IHC)

Lymph nodes from 4 patients with reactive follicular hyperplasia and tissue sections from gastric mucosa and tumors of the thigh, neck and thyroid gland from 4 patients with BL were evaluated. All individuals gave informed consent and the study was approved by the Institute of Hematology and Transfusion Medicine Ethics Committee. Tissue biopsies of the patients were histopathologically examined to establish a diagnosis according to 2008 WHO classification. Tissue biopsies were fixed in 10% formalin, routinely processed and stained with hematoxylin and eosin. Immunohistochemistry was performed using the following antibodies: PRDX1 (Sigma-Aldrich, dilution 1:150, pH = 9.0) and PRDX2 (GeneTex, clone EPR5154, dilution 1:200, pH = 9.0). Staining was performed according to the manufacturer's instructions. The EnVision System (Dako) was used for detection. Positive controls included human tonsils (PRDX1) and human prostatic hyperplasia (PRDX2). The negative (isotype) controls were generated with ready to use FLEX Negative Control Mouse (cocktail of mouse IgG1, IgG2a, IgG2b, IgG3 and IgM; code No IR750; Dako). Samples were reviewed for the expression of lymphoid cells by MPS. Appropriate cellular localization for immunostaining was membrane and cytoplasmic for PRDX1 and PRDX2. All photographs were taken using DP72 Olympus BX63 microscope camera (Olympus, Japan).

### Gene expression analysis

To examine expression levels of the genes involved in the red-ox pathway in the selected populations of B cells we accessed the E-GEOD-12453 data set [22] deposited in the ArrayExpress database [43]. Briefly, the raw (CEL) data was normalized using the robust multiarray average (RMA) procedure using a custom CDF environment [44] in order to collapse the features to the Entrez gene level. The normalized data was then mean-centered and scaled. The significance for each gene to differentiate the respective B cell classes was computed using analysis of variance and the differences in expression between the normal B cells and DLBCL and BL samples, respectively, was done using the Mann-Whitney non-parametric test. All of the statistical procedures were done in R.

### Downregulation of PRDX1 and PRDX2

PRDX1 was downregulated using pLKO.1-puro vector-based lentiviral transduction particles purchased from Sigma-Aldrich, USA. To downregulate PRDX2, plasmids encoding PRDX2-specific shRNA as well as hygromycin resistance gene were purchased from ATCGbio Life Tech Inc., USA. Lentiviruses production and transductions were performed according to manufacturer's instructions. Targeting sequences and experimental details are provided in Supplementary Methods.

## SUPPLEMENTARY FIGURES AND TABLES


