# Supplementary material for: Dimeric peroxiredoxins are druggable targets in human Burkitt lymphoma
Source: Oncotarget. 2015 Nov 30;7(2):1717–31. doi: 10.18632/oncotarget.6435 (PMC4811492; doi:10.18632/oncotarget.6435)
Supplement: Supplementary file 1 [file oncotarget-07-1717-s001.pdf]

SUPPLEMENTARY FIGURES AND TABLES

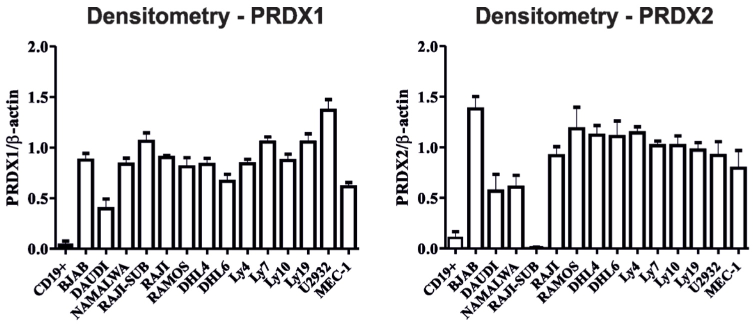

Supplementary Figure S1: Densitometric analysis of 4 blots (mean ± SEM) for PRDX1 and PRDX2. Densitometry data presented is normalized to the levels of β-actin bands using ImageJ software.

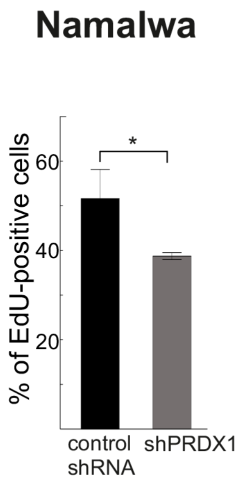

Supplementary Figure S2: PRDX1 knockdown reduces proliferation of Namalwa cells in EdU assay. Data show the % of EdU-positive cells, as described in Methods. Mean values from two independent repeats ± SD, *$P < 0.05$.

A

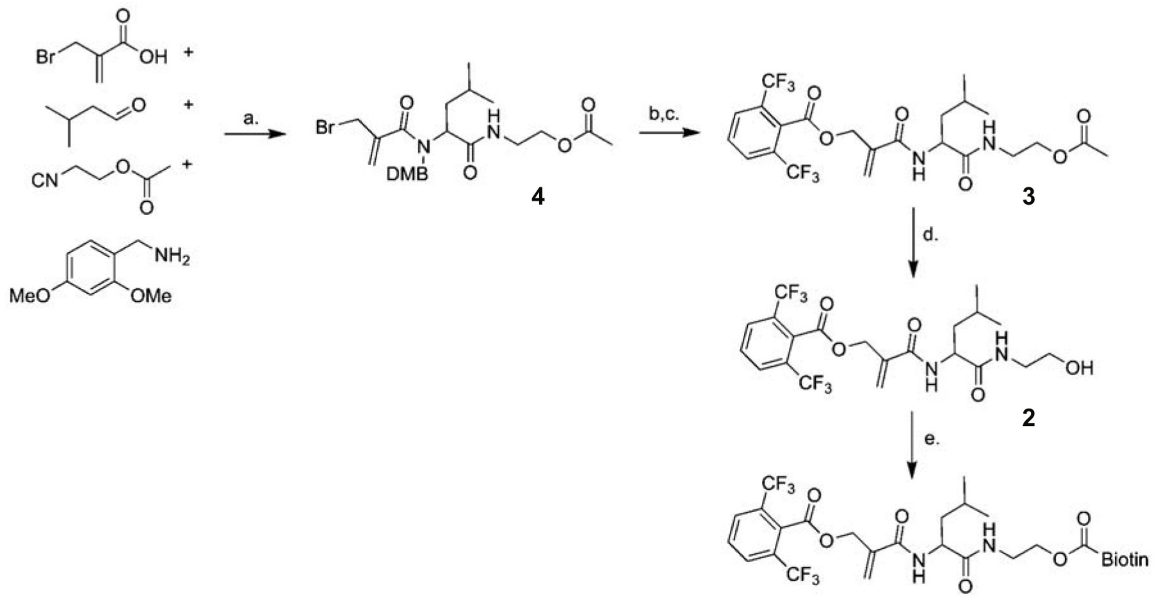

B

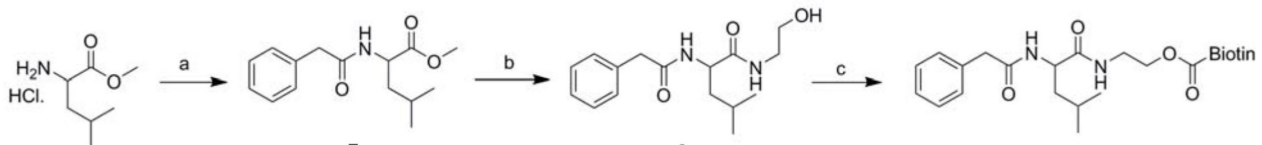

5 6

Supplementary Figure S3: Synthesis of biotinylated analogue of SK053 (SK-bio) and its inactive counterpart (SK-in). A. Synthesis of SK-bio. Reagents and conditions: a) EtOH, 24 h, -78 °C, 67%; b) 20% TFA/DCM, 2 h, RT; c) cesium 2,6-*bis*-(trifluoromethyl)benzoate, acetone, RT, 3 h, 91% (2 steps); d) 2M $K_2CO_{3\ aq}$, MeOH, 15 min, 0 °C , 95%; e) BiotinCOOH, EDC, DMAP, DMF, 28 h, 34%. **B.** Synthesis of SK-in. Reagents and conditions: a) phenylacetic acid, CDI, DCM, 18 h, 64% ; b) ethanolamine, toluene, reflux, 24 h, 64%; c) BiotinCOOH, EDC, DMAP, DMF, 48%.

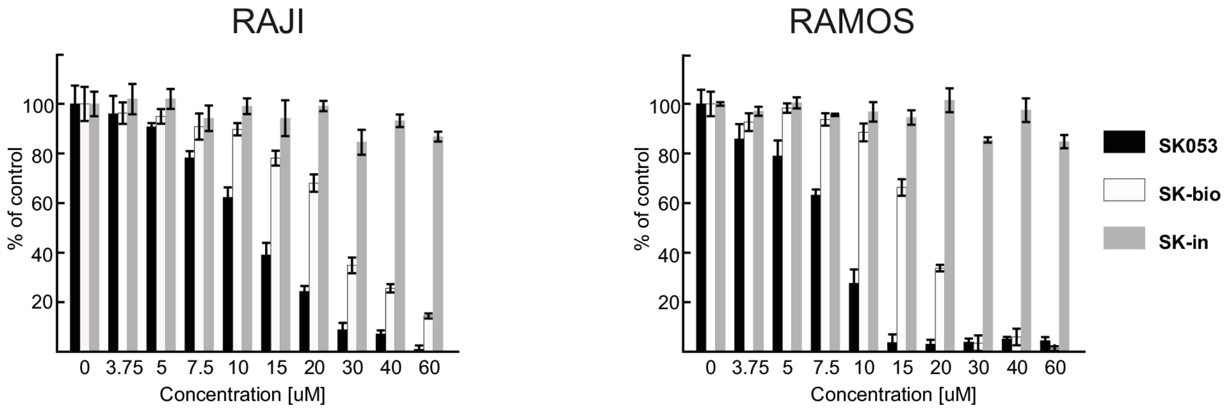

Supplementary Figure S4: Cytotoxic/cytostatic effect of SK053, SK-bio, and SK-in on Raji and Ramos cells. After 48-hour treatment with the compounds, cell viability was assessed by MTT assay, as described in Methods. Results are presented as mean ± SD (*n* = 4).

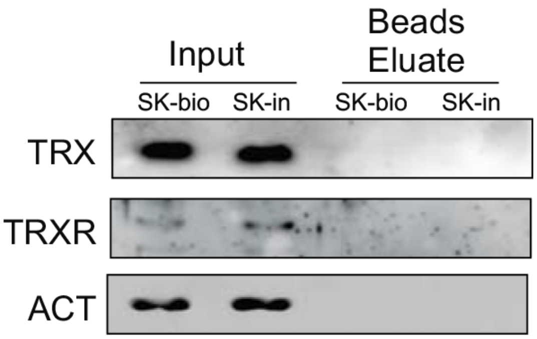

Supplementary Figure S5: Detection of SK-bio-binding proteins by immunoblotting. SK-bio-binding proteins were affinity-purified as described in Figure 3C. and the biotin-eluates were subjected to immunoblotting using antibodies specific to TRX, TRXR, β-actin (ACT).

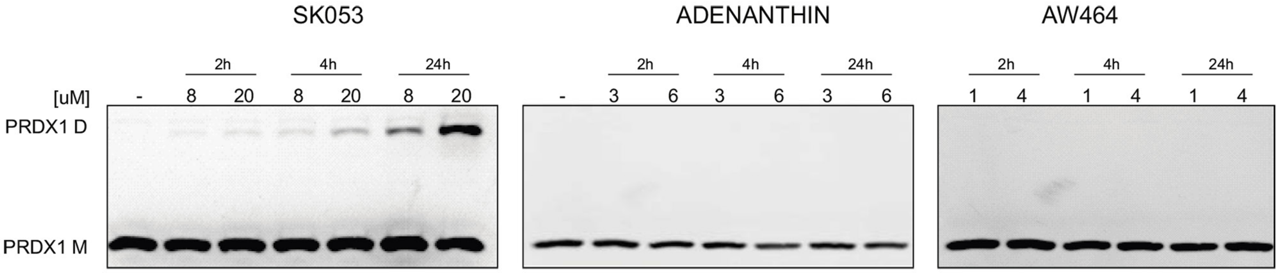

Supplementary Figure S6: 2-Cys PRDXs dimer formation is not a feature of other PRDX or TRX/TRXR inhibitors. Raji cells were treated with equivalent effective doses (~ LD_{50} and LD_{80}) of SK053, PRDX1/2 inhibitor – adenanthin or TRX/TRXR inhibitor – AW464. Cell lysates were subjected to Western blot analysis under reducing and denaturing conditions for detection of covalent PRDX1 dimer formation.

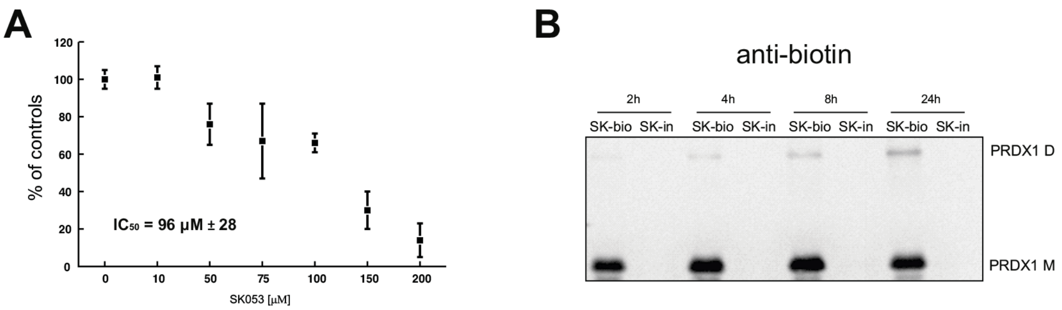

Supplementary Figure S7: SK053 binds recombinant human PRDX1 and inhibits its activity. **A.** The activity of recombinant human PRDX1 was measured in an NADPH-dependent assay. **B.** Binding studies with recombinant human PRDX1 were performed at a protein concentration of 0.1 µg/µl using 20 µM SK-bio/SK-in. After the indicated incubation times, the proteins were subjected to SDS-PAGE in reducing conditions and anti-biotin immunoblot analysis.

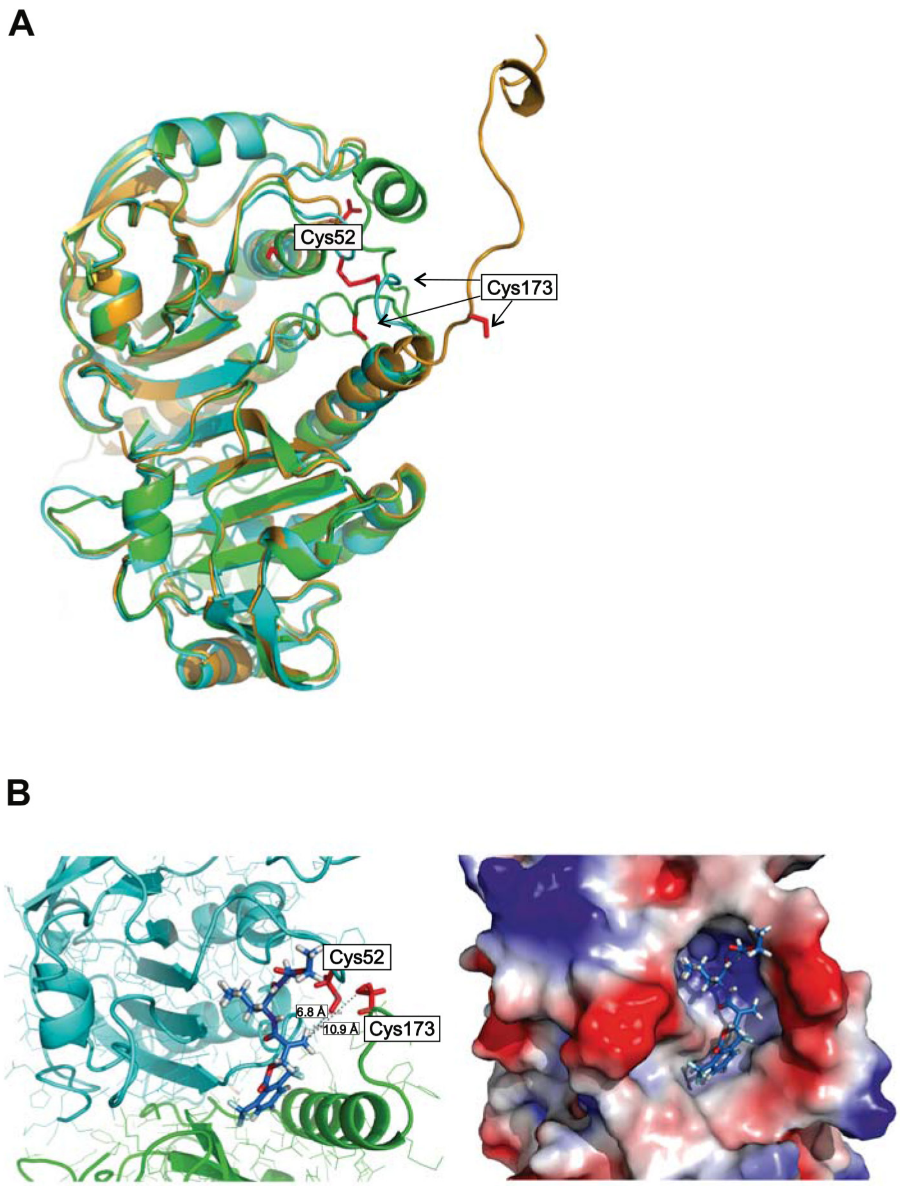

Supplementary Figure S8: Analysis of SK053 binding to PRDX1 structure. A. Superposition of PRDX1 structures in various stages of catalysis: reduced form (PDB code 2Z9S, green), oxidized form (PDB code 1QQ2, blue) and in the complex with sulfiredoxin (PDB code 3HY2, yellow). Active site Cys residues are displayed as red sticks and labeled. Variable positions of Cys173 are indicated with arrows. **B.** Docking of SK053 molecule to the binding pocket around Cys52 of oxidized PRDX1 (PDB code 1QQ2), in which all rat-specific residues were substituted with human equivalents. **Left panel**: Subunits A and B of PRDX1 dimer are displayed in blue and green color, respectively. Active site Cys residues are displayed as red sticks and labeled. Distance between ligand and cysteines is indicated. **Right panel**: Model of PRD1 dimer, in the same orientation, shown in the surface representation, colored according to the distribution of the electrostatic surface potential calculated with Adaptive Poisson-Boltzmann Solver program, blue-positively charged regions, red-negatively charged regions. Graphics are prepared with PyMOL.

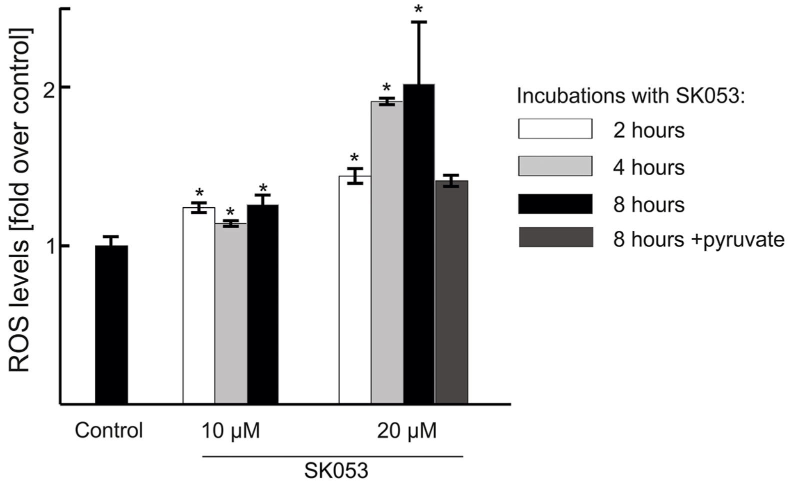

Supplementary Figure S9: Detection of ROS with CellROX fluorescent probe. Raji cells were treated with SK053 for the indicated times and the levels of ROS were detected with CellROX green fluorescent probe (ThermoFisher), according to manufacturer's protocol. The green fluorescence intensity was evaluated by flow cytometry, and the ROS levels are presented as the fold change over untreated control. The mean values from two independent repeats ± SD are shown, $*P < 0.05$.

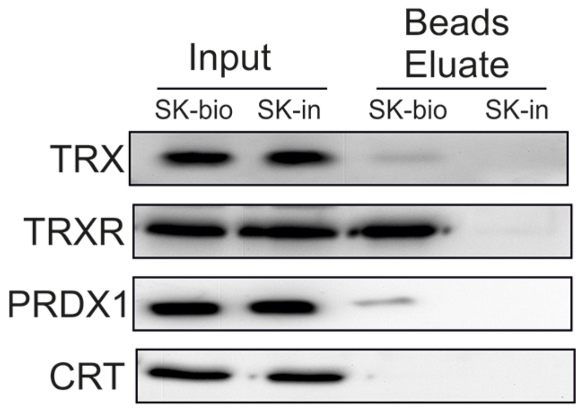

Supplementary Figure S10: Detection of SK-bio-binding proteins in Raji cell lysates by immunoblotting. Raji-sub lysates were incubated with SK-bio or SK-in, and biotin-binding proteins were isolated by neutravidin-affinity pull-down. Subsequently, the proteins eluted from beads were separated by SDS-PAGE and subjected to immunoblotting.

Supplementary Table S1: Analysis of variance results computed for the genes involved in the PRDX-TRX-TRXR antioxidant system

gene	aov.*p*.value
PRDX1	5.19e-10
PRDX2	2.21e-10
PRDX3	1.75e-06
PRDX4	2.10e-15
TRX1	8.30e-14
TRXR1	1.30e-06

The results indicate a pronounced capacity of the genes to differentiate the studied functional classes of B cells.

Supplementary Table S2: Mann-Whitney test results for the comparison between the group of functionally normal B cells and the DLBCL or BL samples respectively

gene	log2fc.DLBCL.vs.normal	p.DLBCL.vs.normal	log2fc.BL.vs.normal	p.BL.vs.normal
PRDX1	1.29	6.15e-05	1.32	8.42e-04
PRDX2	1.00	4.74e-03	2.05	1.40e-05
PRDX3	0.32	8.13e-01	0.44	7.46e-01
PRDX4	1.16	1.93e-03	0.97	3.65e-02
TRX1	1.65	3.33e-09	1.97	1.40e-05
TRXR1	0.49	5.20e-01	0.21	1.00e+00

Supplementary Table S3: Primers for mutagenesis

Mutated residue	Forward primer sequence (5′–3′)	Reverse primer sequence (5′–3′)
Cys52	GACTTCACCTTTGTGGCCCCCACGGAGATCATTG	CAATGATCTCCGTGGGGGCCACAAAGGTGAAGTC
Cys173	CAAACATGGGGAAGTGGCCCCAGCTGGCTGGAAAC	GTTTCCAGCCAGCTGGGGCCACTTCCCCATGTTTG
Cys83	CTTCTGTGGATTCTCACTTCGCTCATCTAGCATGGGTC	GACCCATGCTAGATGAGCGAAGTGAGAATCCACAGAAG

Sites of mutations are underlined.

Supplementary Table S4: Primers for real-time PCR

Name	Sequence
PRDX1 forward	5′-CACTGACAAACATGGGGAAG-3′
PRDX1 reverse	5′-TTTGCTCTTTTGGACATCAGG-3′
PRDX2 forward	5′-GCCTTCCAGTACACAGACGA-3′
PRDX2 reverse	5′-GTTGGGCTTAATCGTGTCACT-3′
B2M forward	5′-TGGAGGCTATCCAGCGTACT-3′
B2M reverse	5′-CGGATGGATGAAACCCAGAC-3′
RPL29 forward	5′-CAGCTCAGGCTCCCAAAC-3′
RPL29 reverse	5′-GCACCAGTCCTTCTGTCCTC-3′

Supplementary Table S5: Target sequences for PRDX1 and PRDX2 downregulation

Name	Sequence
PRDX1 shRNA1	CCATGAACATTCCTTTGGTATC
PRDX1 shRNA2	GCTTTCAGTGATAGGGCAGAA
PRDX2 shRNA	GGTAACCAATAAAGTATTA

SUPPLEMENTARY MATERIALS AND METHODS

Chemical synthesis of biotin-labeled SK053 (SK-bio) and its inactive counterpart (SK-in)

Biotinylated compound SK-bio was obtained from corresponding alcohol **2**. Compound **3** was prepared according to the method applied for SK053 [1]. The compound 2 was prepared as follows: the solution of compound **3** (58 mg, 0.1 mmol) in methanol (1.2 ml) was cooled to 0 °C. Then, the aqueous solution of potassium carbonate was added (2 M, 64 µl). The progress of the reaction was monitored by TLC. After 15 min, methanol was evaporated *in vacuo* without heating. Dichloromethane (10 ml) and the brine (3 ml) were added. Then, the aqueous phase was extracted with dichloromethane (3 × 5 ml). The organic phase was dried over magnesium sulfate, filtered and evaporated. The colorless oil can be used without further purification (51 mg, 95% yield).

Compound **2** (**SK-bio**) (24 mg, 0.05 mmol) was dissolved in DMF (1 ml). Then *D*-biotin (13 mg, 0.05 mmol), DMAP (cat. 2 mg) were added. The solution was cooled to 0 °C and EDC.HCl (10 mg, 0.05 mmol) was added. The reaction was carried out for 28 hours at RT. Then DMF was removed *in vacuo*. Crude product was purified by preparative TLC plates (silica gel, chloroform:methanol 10:1). 12 mg of the biotinylated compound SK231 was obtained as a diastereoisomeric mixture (1:1) with 34% yield. ^{1}H NMR (500 MHz, $CDCl_3$) δ0.81 (d, J = 6.5 Hz, 3H), 0.86 (d, J = 6.5 Hz, 3H), 0.93 (dd, J = 11.3 Hz, J = 6.2 Hz, 6H), 1.37 – 1.44 (m, 2H), 1.45 – 1.59 (m, 6H), 1.59 – 1.71 (m, 8H), 1.71 – 1.81 (m, 4H), 2.19 – 2.28 (m, 2H), 2.31 – 2.36 (m, 2H), 2.69 (d, J = 10.1 Hz, 1H), 2.71 (d, J = 9.8 Hz, 1H), 2.90 (dd, J = 4.9 Hz, J = 2.6 Hz, 1H), 2.93 (dd, J = 4.9 Hz, J = 2.4 Hz, 1H), 3.06 – 3.11 (m, 1H), 3.12 – 3.16 (m, 2H), 3.35 – 3.38 (m, 1H), 3.62 – 3.68 (m, 1H), 3.79 – 3.84 (m, 1H), 3.87 – 3.95 (m, 1H), 4.02 – 4.07 (m, 1H), 4.17 – 4.22 (m, 1H), 4.33 – 4.37 (m, 2H), 4.39 – 4.43 (m, 1H), 4.47 – 4.50 (m, 1H), 4.51 – 4.56 (m, 2H), 4.58 – 4.63 (m, 1H), 5.04 (s, 1H), 5.12 (s, 4H), 5. 79 (s, 1H), 5.82 (s, 1H), 6.14 (s, 1H), 6.13 (s, 1H), 6.98 (t, J = 6.2 Hz, 1H), 7.07 (bs, 1H), 7.08 (bs, 1H), 7.69 – 7.74 (m, 3H), 7.85 (bs, 1H), 7.90 – 7.93 (m, 4H), 8.33 (d, J = 8.2 Hz, 1H) ^{13}C NMR (125 MHz, $CDCl_3$) δppm 21.0, 22.1, 22.8, 22.9, 23.2, 24.9, 25.2, 27.8, 28.0, 28.3, 28.3, 33.3, 33.5, 36.8, 38.8, 39.0, 40.0, 40.7, 41.1, 52.2, 52.8, 53.8, 55.9, 56.3, 60.4, 60.6, 61.7, 62.2, 63.7, 64.2, 65.6, 65.9, 119.9, 122.0, 124.0, 124.2, 124.2, 126.4, 129.0, 129.3, 129.5, 129.8, 130.0, 130.2, 130.2, 130.6, 130.6, 137.2, 137.5, 164.9, 166.5, 166.8, 173.5, 173.6, 173.7, 173.9, 174.2; HR-MS (ESI, [M + Na^+] calculated for: $C_{31}H_{38}N_4O_7F_6SNa$, 747.2258, found, 747.2277.

Compound **5 (SK-in)** was obtained from *L*-Leucine as described before [2]. Compound **6** was prepared as follows: To a solution of compound **5** (290 mg, 1.1 mmol) in toluene (2 ml) the ethanolamine was added (605 mg, 10 mmol). The reaction was carried out at 50 °C for 24 h. Toluene was removed *in vacuo* and the crude product was recrystallized from hexane:ethyl acetate. The 206 mg of white solid was obtained (64% yield). Product **6** (100 mg, 0.34 mmol) was then dissolved in DMF. DMAP (13 mg) and *D*-biotin (92 mg, 0.37 mmol) were added. Then the reaction was cooled to 0 °C and EDC.HCl was added (72 mg, 0.37 mmol). After 24 h DMF was removed *in vacuo*. The crude product was dissolved in chloroform and the organic layer was washed with citric acid (10%, *aq*.) and sodium bicarbonate (8%, *aq*.). The organic layer was washed with brine and evaporated. The product was purified using preparative TLC plates (silica gel, ethyl acetate). 68 mg of colorless oil was obtained (38%) ^{1}H NMR (400 MHz, $CDCl_3$) δ 0.69 (d, J = 6.1 Hz, 3 H) 0.79 (d, J = 5.9 Hz, 3 H) 1.32 - 1.73 (m, 9 H) 1.92 - 2.18 (m, 2 H) 2.61 - 2.74 (m, 1 H) 2.80 - 2.95 (m, 1 H) 3.08 - 3.17 (m, 1 H) 3.17 - 3.29 (m, 1 H) 3.41 - 3.59 (m, 3 H) 3.87 - 4.08 (m, 2 H) 4.27 - 4.37 (m, 1 H) 4.45 - 4.59 (m, 2 H) 5.95 (br. s., 1 H) 7.10 - 7.31 (m, 5 H) 7.57 (br. s., 1 H) 8.23 - 8.43 (m, 2 H)^{13}C NMR (100 MHz, $CDCl_3$) 22.1, 23.2, 24.8, 25.1, 28.1, 28.4, 33.1, 38.7, 40.7, 41.4, 43.0, 52.6, 56.3, 60.6, 62.3, 63.7, 127.1, 128.7, 129.8, 135.7, 164.9, 171.7, 173.9, 174.8; HR-MS (ESI, [M + Na^+] calculated for: $C_{26}H_{38}N_4O_5SNa$, 541.2455, found, 541.2446.

Protein expression and purification

Bacteria were grown in LB medium at 37 °C and protein expression was induced at an OD600 of 0.7–0.9 with 1 mM IPTG (isopropyl β-D-1-thiogalactopyranoside, Fluka) and continued overnight. Bacterial cells were collected by centrifugation, the pellet was resuspended in 10 ml of lysis buffer consisting of CelLytic B (Sigma-Aldrich), 0.2 mg/ml lysozyme, 50 U/ml benzonase, and lysed for 15 min at RT. Cell debris was removed by centrifugation and the supernatant loaded onto the HiTrap FF crude 5-ml Ni Sepharose column (GE Healthcare Life Sciences AB) equilibrated with binding buffer: 20 mM Tris pH 7.5, 300 mM NaCl, 10 mM imidazole, 10 mM β-mercaptoethanol. The protein was eluted using 20 mM Tris pH 7.5, 150 mM NaCl, 10 mM β-mercaptoethanol buffer with imidazole gradient varying from 10 to 400

mM. The protein was further purified with gel filtration chromatography using Superdex75 10/300 GL column (GE Healthcare Life Sciences) equilibrated with 10 mM Tris 7.5, 100 mM NaCl, 10 mM DTT buffer. The nickel affinity chromatography and subsequent gel filtration were performed using Akta Avant 25 operated by Unicorn 6 software (GE Healthcare Life Sciences). The eluted fractions containing PRDX1 were concentrated using VivaSpin 10 kDa concentrator (Sartorius), mixed 1:1 with glycerol and stored at −20°C.

PRDX1 activity assay

The activity of PRDX1 was measured in a NADPH-dependent assay, based on [3]. PRDX1, pre-reduced with TRX-TRXR, was incubated with different concentrations of SK053 for 1 h and dialyzed against reaction buffer to remove unbound inhibitor and exclude the possibility of TRX/TRXR inhibition, and the reaction was initiated with the addition of TRX, TRXR and NADPH. The activity was measured in a final volume of 100 µL containing the following: reaction buffer, 2.8 µM TRX (IMCO), 0.1 µM TRXR (IMCO), 1 mM H_2O_2, 150 µM NADPH and 10 µM PRDX1. Oxidation of NADPH was measured as a change in the absorbance at 340 nm (Asys UV340M spectrophotometer). Data points were measured in triplicate in individual experiments, and error bars represent the SD. The IC_{50} values were evaluated from two independent experiments, with Four Parameter Logistic Standard Curves Analysis, using SigmaPlot software.

In vitro binding experiments

Prior to treatment with the studied compounds, purified PRDX1 was dialysed against buffer containing 25 mM potassium phosphate pH 7.0, 1 mM EDTA, 100 mM $(NH_4)_2SO_4$. Upon dialysis, PRDX1 at a concentration of 0.1 µg/µl was incubated with 20 µM SK-bio or SK-in in the dialysis buffer at 37 °C at the indicated times.

LITERATURE

1. Klossowski, S., et al., Studies toward novel peptidomimetic inhibitors of thioredoxin-thioredoxin reductase system. J Med Chem, 2012. 55: p. 55-67.
2. Su, L., et al., Development of Synthetic Aminopeptidase N/CD13 Inhibitors to Overcome Cancer Metastasis and Angiogenesis. ACS Med Chem Lett, 2012. 3: p. 959-64.
3. Nelson, K.J. and D. Parsonage, Measurement of peroxiredoxin activity. Curr Protoc Toxicol, 2011. Chapter 7: p. Unit7 10.
